# Results from a Phase I Extension Study of Ciliary Neurotrophic Factor in Patients with Macular Telangiectasia Type 2

**DOI:** 10.1016/j.xops.2025.101009

**Published:** 2025-11-14

**Authors:** Lawrence J. Singerman, Jean-Pierre Hubschman, Martin Friedlander, Emily Y. Chew, Catherine Egan, Muna Bitar, Thomas M. Aaberg

**Affiliations:** 1 Retina Associates of Cleveland, Cleveland, Ohio; 2 Department of Ophthalmology, University of California at Los Angeles, Los Angeles, California; 3 Department of Cell and Molecular Biology, The Scripps Research Institute, Division of Ophthalmology, Scripps Clinic, and Lowy Medical Research Institute, La Jolla, California; 4 Division of Epidemiology and Clinical Applications, National Eye Institute, National Institutes of Health, Bethesda, Maryland; 5 NIHR Biomedical Research Centre, Moorfields Eye Hospital NHS Foundation Trust, London, United Kingdom; 6 Neurotech Pharmaceuticals, Inc, Cumberland, Rhode Island; 7 Retina Specialists of Michigan, Foundation for Vision Research, Grand Rapids, Michigan

**Keywords:** Ciliary neurotrophic factor, Long term, Macular telangiectasia type 2, NT-501, Revakinagene taroretcel-lwey

## Abstract

**Objective:**

To primarily assess long-term safety and retinal imaging outcomes of NT-501 (revakinagene taroretcel-lwey), which releases ciliary neurotrophic factor into the vitreous over an extended time, for treating macular telangiectasia type 2 (MacTel).

**Design:**

Phase I, nonrandomized, multicenter, open-label extension study.

**Participants:**

Six participants with bilateral MacTel who completed the parent 60-month phase I study.

**Methods:**

In the parent study, participants had NT-501 surgically implanted in the study eye. The eye with more advanced disease was determined to be the study eye. For the purposes of this extension study, the fellow eye provided untreated natural history data. The extension study included visits 72, 84, 96, and 108 months postimplantation.

**Main Outcome Measures:**

Safety outcomes included adverse events (AEs), change from baseline in best-corrected visual acuity (BCVA), and the proportions of eyes with ≥10- or ≥15-letter loss in BCVA from baseline. Retinal imaging variables included change from baseline in ellipsoid zone (EZ) (inner segment/outer segment) area loss and proportion of study eyes with ≥35% increase from baseline in EZ area loss.

**Results:**

All implants were retained through the final study visit. All ocular treatment-emergent AEs were mild to moderate; none resulted in study discontinuation. No study eyes had ≥15-letter loss in BCVA from baseline at any study visit. Similarly, no study eyes had ≥10-letter loss at months 72, 84, and 96; 1 study eye (17%) experienced it at month 108. The portion of study eyes with a ≥35% increase in EZ area loss from baseline was lower (range, 50%–60%) relative to fellow eyes (range, 75%–100%).

**Conclusions:**

Over the 9-year follow-up period, NT-501 was well tolerated and safe. Further studies are ongoing to investigate the long-term efficacy of NT-501 for treating MacTel.

**Financial Disclosure(s):**

Proprietary or commercial disclosure may be found in the Footnotes and Disclosures at the end of this article.

Macular telangiectasia type 2 (MacTel) is a progressive neurodegenerative retinal disease that leads to vision loss and functional impairment.^1^^,^^2^ The mean age of diagnosis is 57 years of age, with symptoms appearing when people are in their 40s and 50s.^1^^,^^3^ The estimated prevalence of MacTel ranges from 0.005% to 0.1%.^4^, ^5^, ^6^ However, there is low awareness of MacTel among clinicians and patients. In addition, it shares overlapping clinical features with other conditions such as diabetic retinopathy and neovascular age-related macular degeneration, which may lead to misdiagnosis.^1^ Therefore, these rates are likely an underestimation of the true disease prevalence.^1^

Macular telangiectasia type 2 was initially believed to be a vascular condition due to the presence of telangiectatic vessels in the macula.^7^ However, it has been demonstrated to be a neurovasculoglial degenerative condition involving dysfunction of neurons, vasculature, retinal pigment epithelium (RPE) cells, and Müller glia. Accordingly, MacTel is associated with telangiectasis, photoreceptor apoptosis, RPE hyperplasia and disorganization, as well as atrophy of the outer retina.^8^ OCT demonstrates corresponding retinal degenerative hyporeflective cavities.^8^ Previously thought to be a localized retinal condition, it is now known to have a systemic metabolic component involving dysregulation of serine metabolism and leading to abnormalities in patients’ sphingolipid profiles.^9^ While not a classic Mendelian disease, MacTel has a genetic component, and causative mutations involving serine metabolism have been found in 4% to 5% of patients with MacTel.^10^^,^^11^

Patients with MacTel often experience metamorphopsia, scotomas, and loss of vision that interfere with activities of daily living such as reading and driving.^2^^,^^8^^,^^12^, ^13^, ^14^ Macular telangiectasia type 2 can affect people during their prime earning years, leading to an economic burden.^15^ Long-term studies have demonstrated progressive vision loss, with most vision loss in advanced cases due to RPE hyperplasia, the development of macular holes, or choroidal neovascularization.^8^^,^^16^^,^^17^ Macular telangiectasia type 2 can negatively affect patients’ general health, mental health, social functioning, loss of independence, and quality of life.^13^^,^^14^

Due to its protective role within the retina,^18^ ciliary neurotrophic factor (CNTF) has been investigated in the treatment of degenerative diseases such as retinitis pigmentosa, age-related macular degeneration, and primary open-angle glaucoma.^19^, ^20^, ^21^ In preclinical models of retinal degeneration, intravitreal injection of CNTF reduced photoreceptor loss.^22^^,^^23^ Similarly, in vitro studies demonstrated that CNTF significantly increased the survival of purified retinal ganglion cells at low concentrations, with a positive correlation between CNTF concentration and retinal ganglion cell survival rate.^24^^,^^25^ In vivo and clinical studies also indicated that CNTF has a protective effect on retinal ganglion cells and can slow or prevent the progression of photoreceptor cell death in retinal degenerative diseases.^25^, ^26^, ^27^

NT-501, or revakinagene taroretcel-lwey (Neurotech USA), is approved by the US Food and Drug Administration for the treatment of MacTel in adults based on the results of 2 phase III trials.^28^ It is an intravitreal implant composed of a cylindrical membrane that encapsulates RPE cells transfected with the CNTF-producing gene.^29^ The cylindrical membrane is capped on both ends and is semipermeable, allowing for therapeutic proteins (such as CNTF) produced by the genetically modified RPE cells and nutrients to diffuse freely across the membrane while creating a barrier to the host immune system.^30^ After surgical implantation, the implant has been shown to deliver bioactive CNTF over 14 years.^31^

The 36-month interim safety results from an open-label 60-month phase I trial of NT-501 in adult patients with bilateral MacTel have been published.^29^ Overall, NT-501 was well tolerated. No implants required explantation or were extruded over the course of the study, and there were no occurrences of severe intraocular inflammation.^29^ This study was not adequately powered to evaluate the efficacy of NT-501; accordingly, no significant differences in efficacy outcomes were observed.^29^

Herein, we report the results from the 108-month phase I extension study in participants who previously completed the parent phase I study. The primary objective was to investigate the long-term safety and tolerability of NT-501, including changes in best-corrected visual acuity (BCVA). Secondarily, we conducted post hoc analyses to explore changes in the area of ellipsoid zone (EZ) loss for the implanted eye (study eye) and the fellow eye.

## Methods

### Study Design

This study was a phase I, prospective, single-masked, multicenter, extension study that evaluated the long-term safety and retinal imaging outcomes of the NT-501 implant (registered at ClinicalTrials.gov: NCT03071965). The study was planned to include all patients with MacTel who had previously completed 60 months of follow-up in the phase I parent study (registered at ClinicalTrials.gov: NCT01327911).^29^ These studies were conducted at 2 participating sites: the Retina Associates of Cleveland, Inc, and the Jules Stein Eye Institute, University of California, Los Angeles. Enrolled participants signed an informed consent document specific to the extension study. Participant safety was monitored by a Data Safety and Monitoring Committee. The Emmes Corporation (Rockville, MD) served as the study coordinating center. The institutional review board/ethics committee of each site (Western Institutional Review Board and University of California, Los Angeles Institutional Review Board) approved the study protocol. The study adhered to the tenets of the Declaration of Helsinki.

The design, inclusion criteria, exclusion criteria, and surgical protocol of the parent study have been described previously.^29^ In brief, enrolled participants had a diagnosis of bilateral MacTel and were aged ≥21 years. Eligible eyes had BCVA of 20/50 or better. The eye with worse BCVA was chosen as the study eye. If BCVA was the same in both eyes, then the eye with less favorable clinical characteristics (as determined by the principal investigator) was selected as the study eye. Patients who had a history of prior intraocular surgery were excluded. Other exclusion criteria of the parent study included the presence of subretinal neovascularization in either eye or concurrent diagnoses of other ocular diseases such as diabetic retinopathy, uveitis, central serous chorioretinopathy, and pathologic myopia. Participants in the extension study had to have completed 60 months of follow-up in the parent study. There were no additional specific inclusion or exclusion criteria for the extension study.

In the parent study, each participant received the NT-501 CNTF encapsulated cell therapy in the study eye, and the untreated fellow eye provided untreated natural history data, although these eyes were not as severely affected as the study eyes at baseline. The implant was implanted through a 2.0-mm sclerotomy created 3.75-mm posterior to the limbus in the inferotemporal quadrant and anchored with a polypropylene suture. The scleral incision was closed with 9-0 nylon sutures, and proper implant placement was confirmed with indirect ophthalmoscopy at the conclusion of the procedure. There was no additional surgical intervention performed in the extension study.

The extension study consisted of 4 visits conducted at months 72, 84, 96, and 108 after the date of NT-501 implantation. At each study visit, participants underwent bilateral manifest refraction, BCVA testing, intraocular pressure (IOP) measurement, microperimetry, slit lamp biomicroscopy, dilated fundus examination, spectral-domain OCT (SD-OCT), fundus autofluorescence, and color digital fundus photography.

### Outcomes

Long-term safety of NT-501 was assessed by monitoring adverse events (AEs), the results of ophthalmic examinations (slit lamp biomicroscopy, dilated fundus examination, IOP measurement, pupil diameter), and BCVA. Adverse events of interest were defined as those affecting ocular function, different from those expected in the normal course of MacTel, and potentially related to the surgical procedure, NT-501, or CNTF. Safety was also measured with the BCVA analyses that included the mean change from baseline in BCVA and the proportion of eyes with at least a 15-letter or at least a 10-letter loss from baseline in BCVA.

Exploratory retinal imaging variables included the change from baseline in the area of EZ (inner segment/outer segment) loss and the proportion of study eyes with a ≥35% increase from baseline in the area of EZ loss. For measurement of the area of EZ loss, en face SD-OCT scans were transferred electronically to a central reading center, and the area of loss was determined by masked readers. A study conducted at the Duke Reading Center demonstrated strong interobserver reproducibility in measuring EZ area loss, with an intraclass correlation coefficient approaching 1.^32^

### Statistical Analyses

The safety and retinal imaging analyses were conducted using descriptive statistics as these were nonrandomized comparisons between those eyes that received NT-501 and their fellow eyes. All AEs that occurred during the study were documented and coded using the Medical Dictionary for Regulatory Activities (v25.0). All AEs were classified as treatment-emergent AEs (TEAEs) for the purpose of analysis. The measurements taken at the most recent visit prior to the surgical implantation procedure were considered as the baseline. For continuous endpoints, summary statistics were provided for the actual value and for the change from baseline. Dichotomous endpoints were summarized using frequency counts and proportions by visit, along with 95% confidence intervals that were computed using the Clopper–Pearson method. Retinal imaging endpoints were summarized descriptively for study and fellow eyes at each scheduled visit. The statistical software package SAS v9.4 (SAS Institute) was used to perform all data analyses and summaries.

## Results

### Study Participants

Six of 7 participants (86%) who participated in the parent study consented to participate in the extension study, for a total of 6 study eyes and 6 fellow eyes. The seventh participant did not complete the parent study and was not eligible for the extension study. All 6 participants completed the extension study. For 3 participants, the 108-month visit was delayed due to the coronavirus disease 2019 pandemic, and the SD-OCT images for assessment of EZ were not assessed in time for the final analysis. Table 1 summarizes baseline demographic and ocular characteristics. The mean (standard deviation [SD]) age at the time of enrollment in the parent study was 53.8 (5.1) years. The study cohort was 67% female (n = 4) and 33% male (n = 2), and the majority (67%) of participants were White (n = 4).

**Table 1:** Baseline Demographic and Ocular Characteristics for Patients Entering the Extension Phase

By Participant	NT-501 (N = 6)
Female, n (%)	4 (67)
Age (yrs), mean (SD)	53.8 (5.1)
Race, n (%)	
White	4 (67)
Asian	1 (17)
Black or African American	0
Other	1 (17)
Ethnicity, n (%)	
Hispanic or Latino	0

By Eye	NT-501 (N = 6)	Fellow Eye (N = 6)
EZ area loss, mm^2^		
Mean (SD)	0.63 (0.81)	0.70 (0.83)
Median (minimum, maximum)	0.24 (0, 1.86)	0.36 (0, 1.93)
Mean baseline BCVA, ETDRS letters (SD)	75.0 (7.9)	80.5 (7.3)
Snellen equivalent	20/32	20/25

BCVA = best-corrected visual acuity; EZ = ellipsoid zone; NT-501 = revakinagene taroretcel-lwey; SD = standard deviation.

The mean (SD) BCVA at baseline was 75.0 (7.9) letters (Snellen equivalent of 20/30) in the study eyes and 80.5 (7.3) letters (Snellen equivalent of 20/25) in the fellow eyes. The mean (SD) area of EZ loss at baseline was 0.63 (0.81) mm^2^ and 0.70 (0.83) mm^2^ in the study eyes and fellow eyes, respectively. The mean (SD) baseline IOP was 16.3 (2.0) mmHg in the study eyes and 16.5 (2.1) mmHg in the fellow eyes. There were no meaningful differences in baseline ocular characteristics between the study eye and fellow eye groups.

### Safety Outcomes

Table 2 and Table S1, available at www.ophthalmologyscience.org, report ocular AEs observed in the extension study through 108 months after postimplantation. All 6 participants (6 study eyes) retained NT-501 through the final study visit. Ocular TEAEs were reported in 4 study eyes (67%) and 3 fellow eyes (50%). All ocular TEAEs were mild to moderate in severity. There were no serious ocular TEAEs, and no AEs resulted in withdrawal from the study. Only 1 ocular TEAE in 1 study eye (17%) was considered by the investigator to be related to the surgical procedure, which was a suture-related complication (i.e., discomfort caused by the polypropylene fixation suture loosening and eroding through the conjunctiva) that occurred after this extension study concluded (approximately 9.6 years after implantation). This led to explantation of NT-501 about 2 weeks after the event was reported. One study eye had device extrusion with a loop visible in the wound at month 96; however, this was not considered clinically significant by the investigator and did not result in explantation of implant.

**Table 2:** Ocular Adverse Events through 108 Months

By Eye, n (%)	NT-501 (N = 6)	Fellow Eye (N = 6)
≥1 ocular TEAE	4 (67)	3 (50)
Ocular TEAEs by maximum severity		
Mild	2 (33)	1 (17)
Moderate	2 (33)	2 (33)
Severe	0	0
Ocular TEAE by relationship		
Surgery	1 (17)∗	—
NT-501	0	—
CNTF	0	—
Ocular TEAE leading to explantation	1 (17)	0

CNTF = ciliary neurotrophic factor; NT-501 = revakinagene taroretcel-lwey; TEAE = treatment-emergent adverse event.

∗ This ocular TEAE occurred after the final month 108 study visit.

No study or fellow eyes experienced clinically significant ocular AEs of interest, as defined in the protocol, during the study. No eyes had an IOP of ≥21 mmHg or an increase in IOP from baseline of 5 mmHg or more at any study visit. Though the TEAE of miosis was not reported by participants or investigators during the extension study, the measured mean pupil diameter was consistently smaller in study eyes (2.3–3.7 mm across visits) compared with fellow eyes (4.0–4.8 mm across visits). No safety signals were reported from the ophthalmic examinations performed over the 108 months of follow-up.

Five participants (83%) had ≥1 nonocular TEAE during the study, and 2 participants experienced multiple nonocular TEAEs. Two participants had nonocular TEAEs that were severe. These included pain in an extremity, duodenal ulcer, duodenal ulcer perforation, procedural pain, and incarcerated hernia, all occurring in the same participant, and vascular pseudoaneurysm occurring in the other participant. Of these severe events, duodenal ulcer perforation, incarcerated hernia, and vascular pseudoaneurysm were also serious. However, none of the nonocular TEAEs were determined to be related to the surgery, NT-501, or CNTF by the investigator.

**Figure 1:**
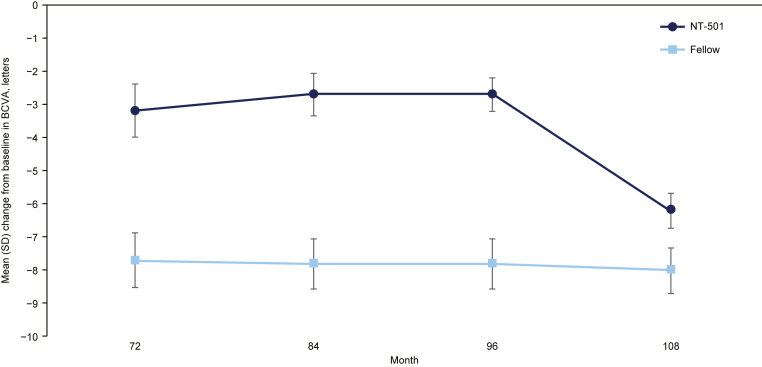
Graph depicting the mean change from baseline in BCVA in eyes that received NT-501 (n = 6) and in fellow control eyes (n = 6) through month 108. Error bars represent SD. BCVA = best-corrected visual acuity; NT-501 = revakinagene taroretcel-lwey; SD = standard deviation.

The mean decrease from baseline in BCVA was consistently less in the study eye group compared with the fellow eye group at each study visit (–3.2 vs. –7.7 letters at month 72; –2.7 vs. –7.8 letters at month 84; –2.7 vs. –7.8 letters at month 96; and –6.2 vs. –8.0 letters at month 108 for study and fellow eyes, respectively) (Fig 1). No study eyes (0%) experienced a ≥15-letter loss in BCVA from baseline at any time point. Two fellow eyes (33%) had a ≥15-letter loss in BCVA from baseline at month 72; no fellow eyes met this criterion at any other time point. Similarly, no study eyes (0%) experienced a ≥10-letter loss in BCVA from baseline at months 72, 84, and 96. One study eye (17%) experienced this level of BCVA loss at month 108. Among the fellow eyes, 2 eyes (33%) had a ≥10-letter loss in BCVA from baseline at 72, 84, 96, and 108 months.

### Rates of Change in EZ Loss in Study and Fellow Eyes

**Figure 2:**
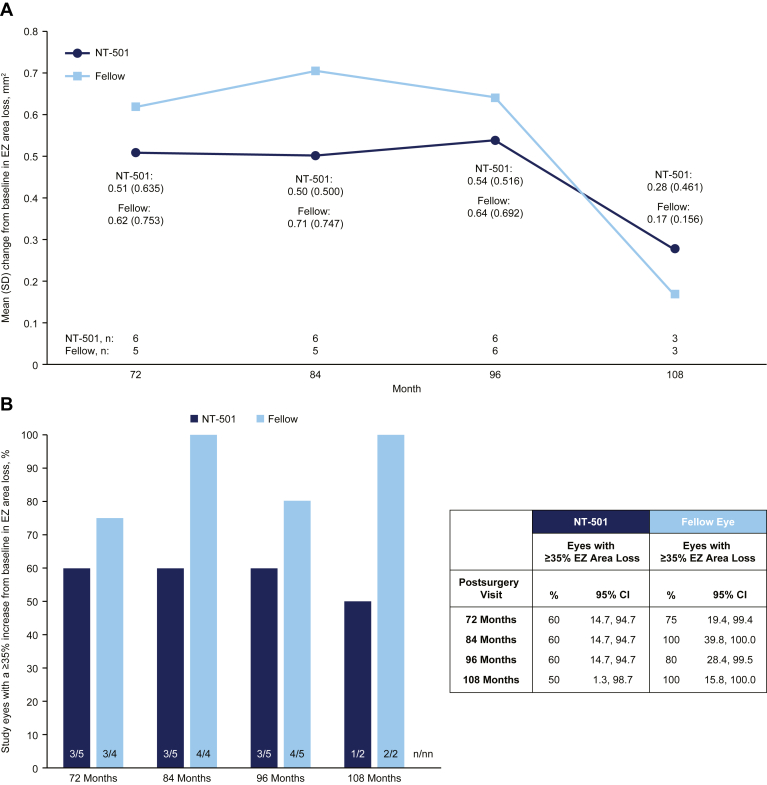
Graphs showing changes from baseline in the area of EZ loss through month 108 in eyes that received NT-501 and in the fellow untreated eyes. **A**, Graph demonstrating mean change from baseline in the area of EZ loss over time, with n representing the number of eyes observed. **B**, Graph showing the percentage of eyes with a ≥35% increase from baseline in the area of EZ loss at each study visit, with n representing the number of eyes with ≥35% increase in EZ area loss and nn representing the number of eyes observed∗. ∗This analysis excludes 1 participant who had a baseline EZ area measurement of 0.0 mm^2^ in both the study eye and the fellow eye for whom a percent change could not be calculated. Their absolute change from baseline to month 108 was 0.03258 mm^2^ in the study eye versus 0.06645 mm^2^ in the fellow eye, representing a 51% reduction in EZ loss progression compared with the fellow eye. CI = confidence interval; EZ = ellipsoid zone; NT-501 = revakinagene taroretcel-lwey; SD = standard deviation.

The mean changes in the area of EZ loss from baseline were consistently lower in the study eyes compared with the fellow eyes at months 72 (0.508 vs. 0.620 mm^2^), 84 (0.503 vs. 0.705 mm^2^), and 96 (0.540 vs. 0.642 mm^2^) (Fig 2A). At month 108, when only 3 participants were evaluated, the mean change in the area of EZ loss from baseline was numerically greater in the study eyes (0.277 mm^2^) compared with the fellow eyes (0.165 mm^2^). There was a lower proportion of study eyes with a ≥35% increase in the area of EZ loss from baseline compared with fellow eyes at all time points (50% to 60% for study eyes vs. 75% to 100% for fellow eyes across all study visits) (Fig 2B). This analysis (≥35% increase in the area of EZ loss from baseline) excludes 1 participant who had a baseline EZ area measurement of 0.0 mm^2^ in both the study eye and the fellow eye for whom a percent change could not be calculated. This participant’s absolute change from baseline to month 108 was 0.03258 mm^2^ in the study eye versus 0.06645 mm^2^ in the fellow eye. No direct comparisons between the study and fellow eyes were evaluated statistically.

## Discussion

This phase I extension study demonstrated that NT-501 is safe and well tolerated in the long term over a follow-up period of 9 years in a small number of eyes with MacTel. All study eyes retained implant through the final visit, although late suture and implant site complications were reported in 2 eyes around the end of this study. All ocular TEAEs were mild or moderate in severity, and none led to discontinuation of the study. No safety signals were observed over the 108 months of post implantation observation. Furthermore, the mean reduction in BCVA from baseline was consistently less in eyes that received NT-501 compared with the untreated fellow eyes at every study visit. No treated eyes had a ≥15-letter reduction in BCVA at any time point, nor did any treated eyes have a ≥10-letter reduction in BCVA through month 96. Among the untreated fellow eyes, 2 eyes had a ≥15-letter BCVA reduction at one time point, and 2 eyes had a ≥10-letter decrease in BCVA at every study time point. The consistently smaller pupil diameter objectively measured in study eyes could reflect the subclinical parasympathomimetic effect of CNTF; however, this degree of pupil constriction may not have met the clinical or functional threshold for miosis or adverse reporting. In this small study sample, we did not identify any signals that NT-501 had any long-term deleterious effect on vision, further supporting its safety.

These safety outcomes are similar to the published results of the parent phase I study, in which NT-501 was well tolerated over a shorter time frame.^29^ No implants were explanted in either the parent study^29^ or the extension study. However, one partial device extrusion occurred at month 96 that was stable when reassessed at month 108 and did not result in explantation, and another participant, 6 months after the study ended (9.6 years after implantation surgery), developed discomfort caused by the polypropylene fixation suture loosening and eroding through the conjunctiva which was managed by explanting the NT-501. In addition, in 24-month results of the published phase II study, visual field, electroretinography, and BCVA outcomes did not reveal any safety concerns in patients treated with the implant.^27^ Taken as a whole, the results of these prior studies are consistent with those of the extension study and support the overall safety of NT-501.^27^^,^^29^

Macular telangiectasia type 2 is a progressive disease, and therefore, BCVA may not be a sensitive enough endpoint to capture early pathological changes or accurately reflect the visual disability these patients experience.^12^ Loss of photoreceptors in patients with MacTel is heterogeneous and by retaining patches of functioning parafoveal neurons, patients can develop eccentric fixation, enabling them to read letters on the eye chart but not necessarily integrate those letters into words while reading. Ellipsoid zone loss on SD-OCT reflects photoreceptor degeneration or dysflectivity secondary to disease progression.^12^^,^^33^ This structural measure correlates with function assessed by retinal sensitivity loss on microperimetry.^27^^,^^32^^,^^34^ Therefore, the change in the area of EZ loss from baseline was selected as an anatomical efficacy outcome that is likely to be more sensitive and objective to early deterioration than BCVA.^1^ The selection of an OCT-based anatomical endpoint parallels recent clinical studies in geographic atrophy secondary to age-related macular degeneration, in which the area of geographic atrophy measured by fundus autofluorescence was deemed acceptable as a primary efficacy outcome by the US Food and Drug Administration.^35^ Similarly, the area of EZ loss has been used as a primary endpoint in clinical trials for MacTel.^27^^,^^28^ The current study was not powered to test efficacy and was not randomized to treatment assignment. Also, the study eye was the most severely affected eye at baseline, so measured differences in progression would be expected. However, the long-term effect of NT-501 appears to follow the same pattern described in the pivotal phase II and III trials.^27^^,^^28^ The increase in the area of EZ loss was consistently lower in treated eyes compared with fellow eyes at each time point through month 96. In addition, a lower proportion of eyes implanted with NT-501 had a ≥35% increase in the area of EZ loss from baseline compared with fellow eyes at every time point through month 108. In the parent phase I study, which was also not powered to detect significant differences in BCVA or EZ loss (or inner segment/outer segment break area), no clinically significant differences in these outcomes were observed.^29^ In the previously reported phase II study, which included 99 eyes of 67 participants randomized to either sham or NT-501 surgery, eyes that received NT-501 had significantly less photoreceptor loss (measured by the area of EZ loss) at 24 months.^27^ Further investigation of these results in larger, long-term studies is warranted.

This study’s major strength is that it provides long-term safety results. However, it has several limitations. As discussed, the small sample size and nonrandomization limits the interpretation of functional and anatomical outcomes; therefore, the efficacy of NT-501 requires further evaluation. The effect of the small sample size is evident in the reduction in EZ area loss over time seen in both study and fellow eyes at time points throughout the study, with this anomaly likely being due to small sample sizes and missing data. A square root transformation of EZ area loss was not performed in the parent study, which may limit the interpretation of these results. The small and relatively homogeneous participant group may also limit generalizability to broader populations. In addition, the progressive nature of MacTel means that changes in BCVA may not be measurable, even over a very long follow-up period as in the current study. The longitudinal data from the MacTel Project’s Natural History Observation Registration Study documented a mean decrease of approximately one letter of visual acuity per year, with a mean follow-up of 4.2 years (range, 1–6 years),^36^ which is consistent with the fellow eye average –8.0 letter loss over 108 months seen in this phase I extension study. This functional limitation can be addressed through the inclusion of anatomical outcomes, such as the area of EZ loss, that are more objective and more sensitive to early pathological changes.

Overall, NT-501 was well tolerated and safe over the 9-year follow-up period of this study, with all implants retained within this time frame. Further studies are under way to investigate the long-term efficacy of NT-501 for the treatment of MacTel.
