# Supplementary material for: Results from a Phase I Extension Study of Ciliary Neurotrophic Factor in Patients with Macular Telangiectasia Type 2
Source: Ophthalmol Sci. 2025 Nov 14;6(2):101009. doi: 10.1016/j.xops.2025.101009 (PMC12919269; doi:10.1016/j.xops.2025.101009)
Supplement: Table S1 [file mmc1.docx]

**Supplementary Material**

**Table S1**. All Ocular Adverse Events* Occurring in the Intent-to-Treat Population, Organized by Eye

| **By Eye, n (%)** | **NT-501 (N = 6)** | **Fellow Eye (N = 6)** |
| --- | --- | --- |
| Dry eye | 1 (17) | 1 (17) |
| Eye irritation | 1 (17) | 1 (17) |
| Allergic conjunctivitis | 1 (17) | 1 (17) |
| Cortical cataract | 1 (17) | 0 |
| Acquired dacryostenosis | 1 (17) | 0 |
| Eye allergy | 1 (17) | 0 |
| Periorbital dermatitis | 1 (17) | 1 (17) |
| Injection site hemorrhage | 1 (17) | 0 |
| Suture-related complication | 1 (17) | 0 |
| Device expulsion | 1 (17) | 0 |

*All events were coded using the Medical Dictionary for Regulatory Activities (MedDRA, version 25.0). NT-501 = revakinagene taroretcel-lwey.
